# Paraxanthine enhances memory and neuroplasticity more than caffeine in rats

**DOI:** 10.1007/s00221-024-06954-0

**Published:** 2024-12-02

**Authors:** Ralf Jäger, Sidney Abou Sawan, Marco Orrú, Grant M. Tinsley, Martin Purpura, Shawn D. Wells, Kylin Liao, Ashok Godavarthi

**Affiliations:** 1 Ingenious Ingredients L.P, Lewisville, TX 75056 USA; 2 Increnovo LLC, Whitefish Bay, WI 53217 USA; 3 Iovate Health Sciences International, Oakville, ON L6M 0H4 Canada; 4 Department of Pharmacology and Toxicology, University of Utah, Salt Lake City, UT USA; 5 Department of Kinesiology and Sport Management, Texas Tech University, Lubbock, TX USA; 6 Radiant Research Services Pvt. Ltd, Bangalore, 560058 India

**Keywords:** Nootropics, Learning, Cognition, Caffeine, Paraxanthine, BDNF

## Abstract

Paraxanthine (PXN) is the main metabolite of caffeine (CAF). PXN supplementation has been shown to increase measures of cognition, memory, reasoning, response time, and sustained attention; however, no preclinical study has compared the effects of PXN with those of CAF. The aim of this study was to compare the effects of PXN and CAF on memory and related biomarkers in rats. The effects of two different doses of PXN (PXN LOW, PXN HIGH), CAF (CAF HIGH), and a control group on cognition (escape latency in the Morris water maze test), neurotransmitters (acetylcholine, dopamine, and gamma-aminobutyric acid), and neurochemicals (BDNF, catalase, glutathione, and cyclic GMP) were analyzed from whole brain samples in young (8 weeks old) and aged (16 months old) rats. Compared to the control group, escape latency improved in PXN LOW, PXN HIGH, and CAF HIGH (all *P* < 0.05) in young animals, and in PXN HIGH and CAF HIGH in older animals (*P* < 0.001). PXN HIGH improved escape latency compared to CAF HIGH in both young (*P* < 0.001) and old animals (*P* = 0.003). BDNF levels increased in PXN LOW, PXN HIGH, and CAF HIGH (all *P* < 0.001), with PXN HIGH increasing BDNF to a greater extent compared to CAF HIGH (*P* = 0.03). PXN HIGH also significantly increased BDNF levels compared to PXN LOW (*P* < 0.001). All other neurotransmitters and neurochemicals significantly increased in the PXN HIGH and CAF HIGH groups compared to the control. In conclusion, PXN showed greater improvements in cognition and BDNF levels compared to CAF, further substantiating PXN as a nootropic with greater benefits compared to CAF.

**Supplementary Information:**

The online version contains supplementary material available at 10.1007/s00221-024-06954-0.

## Introduction

Caffeine (CAF) is one of the most consumed naturally occurring nootropics and exercise-related ergogenic aids (Guest et al. 2021). However, studies on CAF supplementation have shown substantial variability in outcomes, with some studies reporting no benefits or even a worsening of performance in certain subjects. Individuals with a homozygous A allele of the CYP1A2 gene tend to produce more cytochrome P450, an enzyme responsible for about 95% of caffeine metabolism, and consequently metabolize CAF more quickly. Fast metabolizers of CAF experience greater ergogenic outcomes in most studies (Guest et al. 2018; Minaei et al. 2022; Womack et al. 2012), but not all (Pataky et al. 2016), indicating that the metabolic products of CAF are the actual active ingredients.

Paraxanthine (1,7-dimethylxanthine, PXN) is the main metabolite of CAF, accounting for 70–72% of ingested CAF and 85% of the methylxanthine metabolic by-products (Stavric 1988). In comparison to CAF, PXN has a shorter half-life (Lelo et al. 1986), is less toxic (Purpura et al. 2021), and is less anxiogenic (Benowitz et al. 1995). Like CAF, PXN is a central nervous system stimulant; however, compared to CAF, PXN has higher binding potency for adenosine A1 and A2A receptors and produces more substantial locomotor activation effects (Orrú et al. 2013). PXN inhibits phosphodiesterase 9 (PDE9), which terminates nitric oxide (NO) neurotransmission by metabolizing cyclic guanosine monophosphate (cGMP) back to GMP. Through PDE9 inhibition, PXN potentiates NO neurotransmission, an effect that is absent with CAF (Orrú et al. 2013; Ferré et al. 2013).

Similar to CAF (Canas et al. 2009; Dall’Igna et al. 2003), PXN has been shown to have protective effects on dopaminergic neurons and is reported to protect against neurodegeneration and the loss of synaptic function (Guerreiro et al. 2008). Epidemiological studies suggest that higher intake of CAF reduces the risk of Alzheimer’s disease (Eskelinen and Kivipelto 2010). Preclinical studies indicate that reduced β-amyloid production is the likely mechanism behind caffeine’s protective effects on cognition (Arendash et al. 2006). CAF has been shown to improve memory by increasing brain-derived neurotrophic factor (BDNF) levels in mice (Sallaberry et al. 2013).

Beyond these mechanisms, broader evidence indicates that the impact of CAF on memory is intricately linked to A2A receptor-mediated control of synaptic dysfunction. This association has been observed in various models, including non-amyloid models of Alzheimer’s disease (Viana da Silva et al. 2016; Espinosa et al. 2013; Gonçalves et al. 2019; Laurent et al. 2014), as well as in animal models not characterized by altered amyloid metabolism, such as instances of repeated stress (Kaster et al. 2015), convulsions (Cognato et al. 2010), diabetic encephalopathy (Duarte et al. 2012), and aging (Temido-Ferreira et al., 2022). Thus, the neuroprotective and memory-preserving effects of CAF have been consistently associated with the correction of synaptic dysfunction. However, a direct comparison between CAF and PXN regarding their impact on cognition and associated mechanisms is lacking.

Aging is accompanied by a range of physiological and neurological changes that can significantly impact cognitive function, including declines in synaptic plasticity (deToledo-Morrell et al., 1988), neurogenesis (Culig et al. 2022), and alterations in neurotransmitter systems (Mora et al. 2007)—all of which may impair learning and memory. Clinical studies in young (Lieberman et al. 1987; Kaplan et al. 1997) and older adults (Ryan et al. 2002; Rees et al. 1999) generally demonstrate that CAF enhances attention, alertness, and short-term memory at dosages ranging from approximately 32 to 500 mg. PXN, a less-studied metabolite of CAF, has distinct pharmacokinetic and pharmacodynamic properties (Lelo et al. 1986; Benowitz et al. 1995), increasing dopaminergic activity (Guerreiro et al. 2008) and potentially improving memory consolidation more effectively than caffeine. However, the extent of these benefits across different age groups, particularly regarding age-related cognitive decline, remains to be explored.

We recently reported that acute ingestion of PXN enhanced memory, reaction time, and attention for up to six hours in healthy adults (Yoo et al. 2021), and that acute ingestion of as little as 50 mg of PXN for seven days enhanced measures of cognition, memory, reasoning, response time, and sustained attention (Xing et al. 2021). However, no preclinical study has directly compared the cognitive effects of PXN and CAF supplementation. The primary aims of this study were to determine: (1) whether PXN ingestion affects cognitive function in young and old rats; (2) whether PXN is more effective than CAF; and (3) whether CAF and PXN influence the same mechanistic targets known to enhance cognition: neurotransmitter levels, β-amyloid production, BDNF, and antioxidant capacity.

## Materials and methods

### Animals and study design

Sixty-four male Swiss Albino rats, aged 8 weeks (*n* = 32; 8 per group) and 16 months (*n* = 32; 8 per group), were housed in an animal room at a constant temperature (22 ± 3 °C) and humidity (30–70%) under a 12:12 h light-dark cycle, with a standard laboratory diet (Purina 5L79, Rat and Mouse 18% protein; PMI Nutrition International, Brentwood, MO, USA). Animals were housed in standard polypropylene cages (4 animals per cage) with a stainless-steel top grill, containing pelleted food and drinking water. Sterile corncob was used as bedding material and was changed daily. Reverse osmosis-purified water was provided ad libitum. All procedures involving animals were conducted humanely and were performed by or under the direction of trained personnel. The study protocol was reviewed and approved by the Institutional Animal Ethical Committee (IAEC) of Radiant Research Services Pvt. Ltd (Bangalore, India).

To compare the effects of paraxanthine against caffeine and the impact of age, young 8-week-old (YOUNG) and aged 16-month-old (OLD) rats were evenly divided into four groups (*n* = 8 per group): (i) vehicle control (CON), (ii) low-dose paraxanthine (PXN LOW), (iii) high-dose paraxanthine (PXN HIGH), and (iv) high-dose caffeine (CAF HIGH). The doses administered to the rats were calculated using the US Food and Drug Administration guidelines for human equivalence doses (HED), assuming a human weight of 60 kg (Nair et al., 2016). The following HEDs were used in this study: 25 mg/d paraxanthine (PXN LOW; rat dose: 2.57 mg/kg/d), 100 mg/d paraxanthine (PXN HIGH; rat dose: 10.28 mg/kg/d), and 100 mg/d of caffeine (CAF; rat dose: 10.28 mg/kg/d). Paraxanthine (enfinity^®^) was supplied by Ingenious Ingredients L.P. (Lewisville, TX, USA), and caffeine in anhydrous form was supplied by NNB Nutrition, China. Previous studies at a dosage of up to 10 mg/kg caffeine showed no anxiolytic or anxiogenic effects in an open-field test and an elevated plus-maze test (Bhattacharya et al. 1997), nor did it produce either rewarding or aversive effects in a conditioned place preference test (Brockwell et al. 1991). However, it did have a significant facilitative effect on fear extinction (Ozawa et al. 2022).

The prescribed doses were prepared daily by taking the required quantity of the test item in a mortar and pestle. The doses were triturated, and an adequate quantity of vehicle was added, mixed well, and transferred to a volumetric flask. An additional quantity of vehicle was added to the beaker, rinsed, and transferred to the volumetric flask. The required volume was made up by adding sufficient vehicle to the volumetric flask, mixed well, and then transferred to labeled beakers with magnetic beads. Homogeneity of the test item formulations during dose administration was maintained by continuous stirring using a magnetic stirrer. The amount of the test item and the volume of the formulations prepared varied depending on the requirements and/or body weight of the animals. A total of 0.5% carboxymethyl cellulose sodium was used as the CON, and the test item formulations were prepared daily.

Dosing of all test materials was conducted via oral gavage using disposable polypropylene syringes with sterilized stainless steel gavage tubes. The test item formulation was administered once each day by oral route for 11 consecutive days between 9:00 and 11:00 h. The dose volume administered to each rat was 10 mL/kg/day. The dose volume was calculated for individual animals on the first day of treatment and was re-calculated according to the most recent body weights recorded during the treatment period. Body weight was recorded at baseline and on days 5, 8, and 15.

### Morris water maze test

The Morris water maze test is widely used to study spatial memory and learning (Nunez 2008) and was adapted from Morris (Morris 1984). The Morris water maze pool (Orchid Scientific & Innovative India Pvt Ltd) consisted of a circular pool with an inner diameter of 183 cm and an outer diameter of 76 cm in height. The pool was located in a room with geometric shapes on the walls serving as spatial cues. A platform was placed in one quadrant of the pool, submerged 1 cm below the water surface. The water in the pool (25 ± 1 °C) was mixed with non-toxic black paint to prevent the animals from seeing the platform, encouraging them to rely on external maze cues. All animals were trained to locate the submerged platform in a constant location. As the animals became more familiar with the task, they were able to find the platform more quickly, highlighting their spatial memory and learning abilities.

Rats were placed in the water, and escape latency—the time (in seconds) taken to recognize the submerged platform in the designated quadrant—was measured. During training, each rat started at one of four starting points (north, south, east, or west) and was allowed to swim until it located the platform or until 60 s had elapsed. If the rat left the platform within 15 s, the measurement continued. Prior to any supplementation, all animals were subjected to the Morris water maze test to familiarize them with the task. Familiarization was completed over four consecutive days (Days 1–4), with each day providing exposure to each of the four starting points: north, south, east, and west. After the initial training, a probe test was conducted by hiding the platform to ensure that the animals’ performance was truly dependent on spatial memory. Once familiarization was complete, the animals were treated for 10 consecutive days (Days 5–14) in a randomized, placebo-controlled manner. On Day 15, 30 min after administration of the treatments, the animals performed the Morris water maze test. Escape latency was recorded, and the mean of the four starting points was used for data analysis.

### Sample collection and neurochemical analysis

On Day 16, after administration of the final treatment, all animals were euthanized using 95% CO2, and the brain was excised and weighed. The brain tissues were rinsed with ice-cold PBS (pH 7.4) to thoroughly remove excess blood and then weighed before homogenization. A portion of brain tissue (100 mg) was taken in a 900-microliter volume of PBS (1:9 ratio) in a glass homogenizer on ice and homogenized at 2,000 rpm for 5 min. After homogenization, the supernatant was collected for analysis using standard ELISA assay kits according to the manufacturer’s recommendations for clinical biochemical markers, including acetylcholine (BT LAB, China; Lot No.: E0698Mo), dopamine (BT LAB, China; Lot No.: E0667Mo), brain-derived neurotrophic factor (BDNF; Elabscience, USA; Lot No.: E-EL-R1235), β-amyloid (1–40) (Elabscience, USA; Lot No.: E-EL-R3030), catalase (Elabscience, USA; Lot No.: E-BC-K031), glutathione (Elabscience, USA; Lot No.: KTE101106), gamma-aminobutyric acid (GABA; Fine Test, China; Lot No.: ER1707), and cyclic GMP (Fine Test, China; Lot No.: ER0831).

### Statistical analysis

Brain weight, escape latency, and biochemical markers were analyzed using a two-way analysis of variance (ANOVA), with group and age specified as between-subjects factors. Body mass data were analyzed separately for young and aged individuals due to the known differences in body mass between these groups, using two-way ANOVA with group as a between-subjects factor and time (day) as a within-subjects factor. Statistically significant effects were followed up with pairwise comparisons using the Tukey adjustment to account for multiple comparisons. Due to the large number of ANOVA tests, P-values for main effects and interactions were corrected using the false discovery rate method. Partial eta-squared effect sizes were calculated for all ANOVA tests. Homogeneity of variances was tested using Levene’s test, and normality of residuals was examined through visual inspection of quantile-quantile plots. For models with within-subject factors (i.e., the body mass models), the Greenhouse-Geisser correction was applied if sphericity violations occurred. After P-value adjustments, statistical significance was accepted at *P* < 0.05. Data analysis was performed using R (v. 4.2.1). Values are presented as means ± SD (Table.1).

**Table 1:** Summary of statistical results

Variable	ANOVA Term	DF (numerator)	DF (denominator)	F-value	*P*-value (adjusted)	Effect Size
Escape Latency	Group	3	56	931.00	< 0.001*	0.98
	Age	1	56	164.79	< 0.001*	0.75
	Group x Age	3	56	8.00	< 0.001*	0.30
Body Weight (Young)	Group	3	28	1.01	0.60	0.10
	Day	2.4	68.4	9053.44	< 0.001*	1.00
	Group x Day	7.3	68.4	0.57	0.90	0.06
Body Weight (Old)	Group	3	28	0.96	0.60	0.09
	Day	1.5	43.2	1258.21	< 0.001*	0.98
	Group x Day	4.6	43.2	0.58	0.85	0.06
Brain Weight	Group	3	56	0.33	0.90	0.02
	Age	1	56	443.26	< 0.001*	0.89
	Group x Age	3	56	0.17	0.94	0.01
Acetylcholine	Group	3	56	33.96	< 0.001*	0.65
	Age	1	56	86.44	< 0.001*	0.61
	Group x Age	3	56	0.31	0.90	0.02
Dopamine	Group	3	56	104.68	< 0.001*	0.85
	Age	1	56	109.79	< 0.001*	0.66
	Group x Age	3	56	0.46	0.85	0.02
BDNF	Group	3	56	72.74	< 0.001*	0.80
	Age	1	56	58.46	< 0.001*	0.51
	Group x Age	3	56	0.93	0.60	0.05
Amyloid Beta	Group	3	56	65.39	< 0.001*	0.78
	Age	1	56	381.70	< 0.001*	0.87
	Group x Age	3	56	0.55	0.84	0.03
Catalase	Group	3	56	166.94	< 0.001*	0.90
	Age	1	56	17.34	< 0.001*	0.24
	Group x Age	3	56	0.64	0.79	0.03
Glutathione	Group	3	56	200.02	< 0.001*	0.91
	Age	1	56	132.44	< 0.001*	0.70
	Group x Age	3	56	12.98	< 0.001*	0.41
GABA	Group	3	56	34.40	< 0.001*	0.65
	Age	1	56	108.44	< 0.001*	0.66
	Group x Age	3	56	0.13	0.94	0.01
Cyclic GMP	Group	3	56	148.91	< 0.001*	0.89
	Age	1	56	236.52	< 0.001*	0.81
	Group x Age	3	56	0.22	0.94	0.01

*Denotes significant difference

## Results

### Effect of paraxanthine, caffeine and age on body and brain weight

Body weight increased on days 5, 8, and 15 (time effect and pairwise comparisons; *P* < 0.001) in both young and old animals (Table 2) and was not affected by supplement status (group effect; *P* = 0.59).

**Table 2:** Effect of paraxanthine and caffeine on body weight (in grams)

	YOUNG				OLD			
Day	CON	PXN LOW	PXN HIGH	CAF HIGH	CON	PXN LOW	PXN HIGH	CAF HIGH
0	162.9 ± 1.7	163.7 ± 1.4	164.0 ± 1.5	163.8 ± 1.8	490.6 ± 2.0	491.3 ± 2.1	490.3 ± 2.3	489.6 ± 2.1
5*	168.0 ± 1.7	168.6 ± 1.6	169.2 ± 1.2	168.9 ± 1.8	493.6 ± 2.2	494.4 ± 2.2	493.4 ± 2.5	492.6 ± 2.1
8*	171.0 ± 1.7	171.6 ± 1.6	172.4 ± 1.2	171.9 ± 1.8	495.1 ± 2.1	495.8 ± 2.0	494.7 ± 2.4	493.9 ± 2.1
15*	182.4 ± 1.4	183.0 ± 1.5	184.0 ± 1.9	183.2 ± 1.7	500.0 ± 2.2	500.2 ± 2.6	499.0 ± 2.3	498.7 ± 1.9

*Denotes significant difference from day 0 on pairwise comparisons (*P* < 0.001 for each comparison). Data presented as mean ± SD for *n* = 8 in each group. CON, control; PXN LOW, low dose paraxanthine; PXN HIGH, high dose paraxanthine; CAF HIGH, high dose caffeine

Irrespective of supplement status, brain weight was greater in old compared to young animals (age effect; *P* < 0.001) with no effect of group (*P* = 0.92; Table 3).

**Table 3:** Effect of paraxanthine and caffeine on brain weight (in grams)

YOUNG				OLD*			
CON	PXN LOW	PXN HIGH	CAF HIGH	CON	PXN LOW	PXN HIGH	CAF HIGH
1.72 ± 0.03	1.72 ± 0.05	1.72 ± 0.03	1.73 ± 0.03	1.97 ± 0.05	1.97 ± 0.05	1.98 ± 0.07	1.99 ± 0.06

*Denotes main effect of age (*P* < 0.001) with brain weight is greater in OLD compared to YOUNG. Data presented as mean ± SD for *n* = 8 in each group. CON, control; PXN LOW, low dose paraxanthine; PXN HIGH, high dose paraxanthine; CAF HIGH, high dose caffeine

### Effect of paraxanthine, caffeine and age on escape latency

**Fig. 1:**
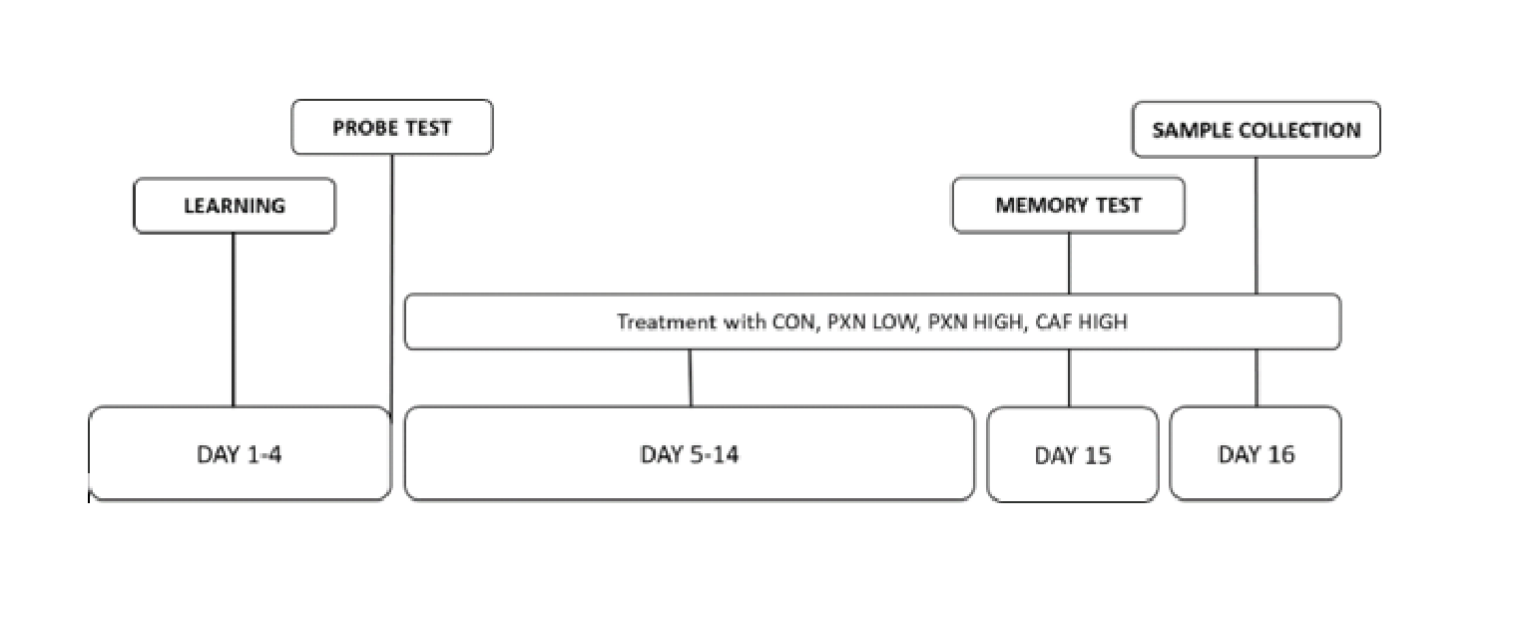
Timeline diagram of the chronological order of manipulations

A group × age interaction was observed for escape latency (*P* < 0.001; Fig. 1). Post hoc analysis revealed that, in young animals, PXN LOW (*P* = 0.049), PXN HIGH (*P* < 0.001), and CAF HIGH (*P* < 0.001) reduced escape latency by approximately 6–60% compared to control. Escape latency was also reduced in CAF HIGH and PXN HIGH by approximately 57% and 50%, respectively, when compared to PXN LOW (both *P* < 0.001). Additionally, in young animals, escape latency was further reduced in PXN HIGH compared to CAF HIGH by approximately 14% (*P* = 0.01).

In old animals, both PXN HIGH (*P* < 0.001) and CAF HIGH (*P* < 0.001) reduced escape latency by approximately 40–47% compared to control and by approximately 37–45% compared to PXN LOW. Compared to CAF HIGH, PXN HIGH reduced escape latency in old animals by approximately 12.1% (*P* = 0.003). No significant differences between control and PXN LOW were observed in aged animals, although a trend was present (*P* = 0.07). Escape latency in the control group for old animals was significantly higher compared to young animals (*P* < 0.01).

### Effect of paraxanthine, caffeine and age on neurochemicals

#### Neurochemicals

**Fig. 2:**
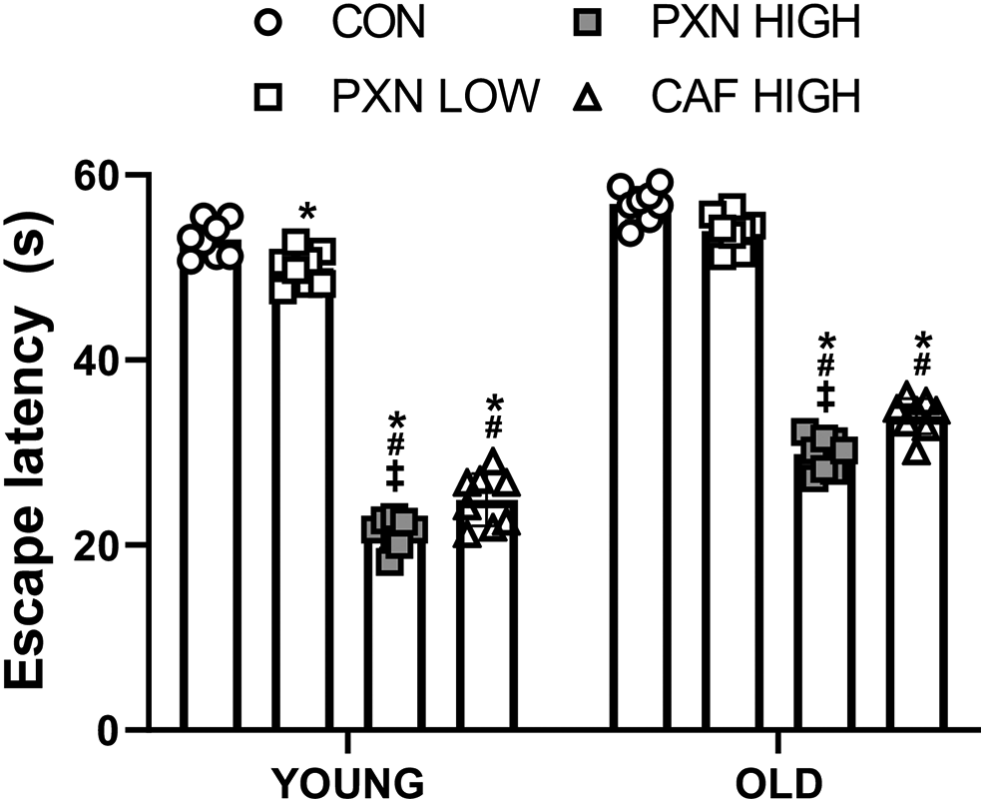
The impact of age and supplementation on learning and memory assessed as highlighted by reductions in escape latency. *Different from CON within same age group (*P* < 0.049). #different from PXN LOW within same age group (*P* < 0.001). ‡Different from CAF within same age group (*P* < 0.01). P-values: Group<0.001; Age<0.001; Interaction<0.001. Data presented as mean ± SD for *n* = 8 in each group. CON, control; PXN LOW, low dose paraxanthine; PXN HIGH, high dose paraxanthine; CAF HIGH, high dose caffeine.

BDNF, catalase, and cyclic GMP levels were higher in animals supplemented with PXN HIGH and CAF HIGH compared to PXN LOW and control (group effect and pairwise comparisons; all *P* < 0.001; Fig. 2A-C). PXN LOW also increased BDNF and catalase compared to control (group effect and pairwise comparisons; all *P* < 0.001). Additionally, PXN HIGH further augmented BDNF (Fig. 2A) to a greater extent compared to CAF HIGH (pairwise comparison; *P* = 0.03).

Both PXN HIGH and CAF HIGH lowered β-amyloid levels (Fig. 2D) compared to PXN LOW and control (group effect and pairwise comparisons; all *P* < 0.001). Finally, except for β-amyloid, young animals had higher levels of all neuromodulators compared to old animals (age effects; all *P* < 0.001).

#### Neurotransmitters

**Fig. 3:**
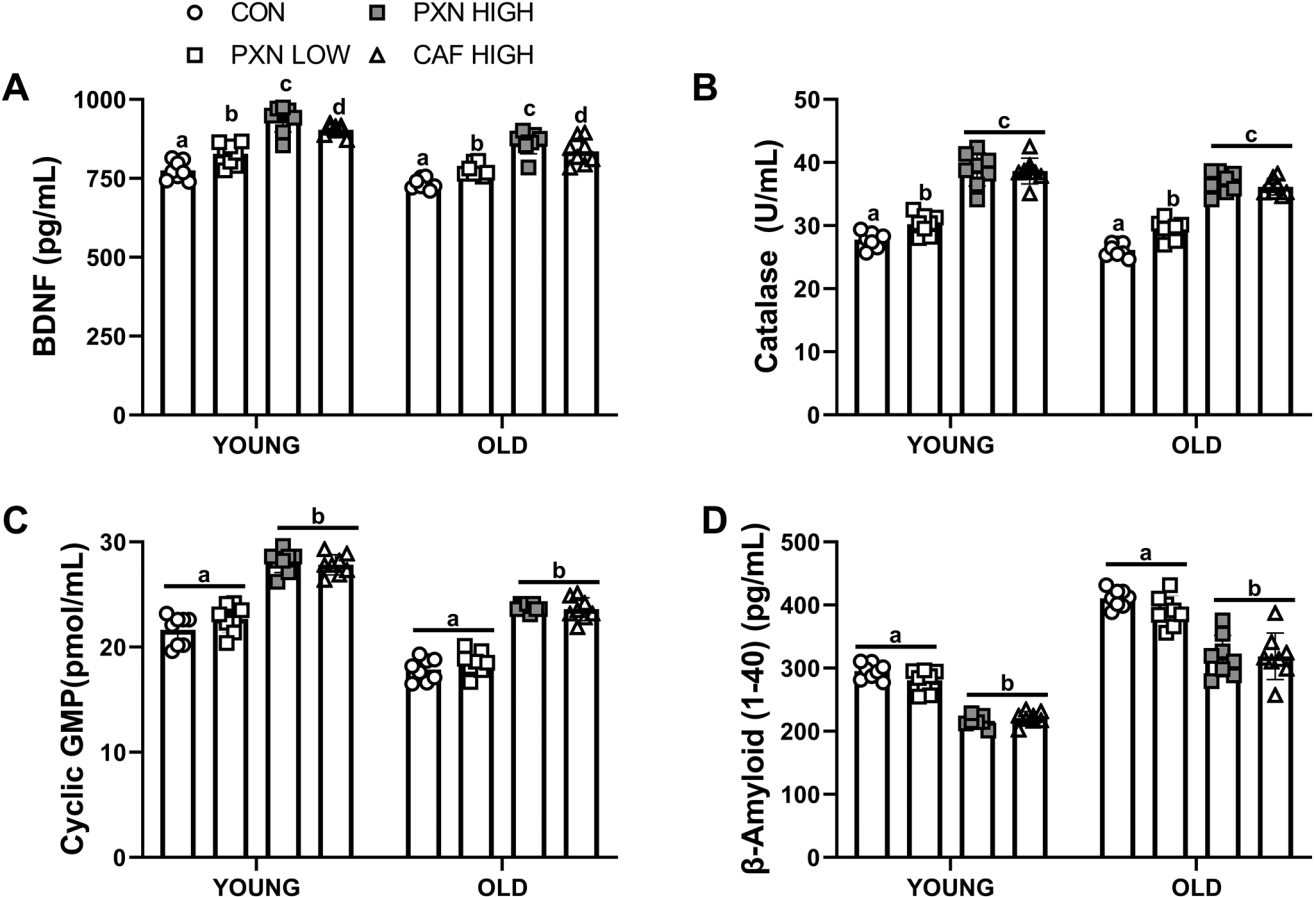
The impact of age and supplementation on BDNF (**A**), catalase (**B**), cyclic GMP (**C**) and β-amyloid (**D**). Letters that are different indicate significantly different group values based on pairwise comparisons following significant group effects (*P* < 0.001). P-values: Group all < 0.001; Age all < 0.001; Interaction < 0.59. Data presented as mean ± SD for *n* = 8 in each group. BDNF, brain-derived neurotrophic factor; CON, control; PXN LOW, low dose paraxanthine; PXN HIGH, high dose paraxanthine; CAF HIGH, high dose caffeine.

Levels of acetylcholine, dopamine, GABA, and glutathione were higher in animals supplemented with PXN HIGH and CAF HIGH compared to PXN LOW and control (group effect and pairwise comparisons; all *P* < 0.001; Fig. 3A-D). In both young and old animals, glutathione (Fig. 3D) was elevated in the PXN HIGH and CAF HIGH groups compared to PXN LOW and control (group × age interaction and pairwise comparisons; *P* < 0.001). Additionally, in old animals, glutathione levels were greater in PXN LOW compared to control (*P* = 0.02). Young animals supplemented with PXN HIGH and CAF HIGH had higher glutathione levels compared to old animals (*P* < 0.01). Finally, young animals exhibited higher levels of all neurotransmitters compared to old animals (group × age interaction and pairwise comparisons; *P* < 0.001).

## Discussion

We aimed to investigate the effects of paraxanthine and caffeine on learning and memory, as well as to assess neurochemical changes in young and old animals. Our findings show for the first time that both paraxanthine and caffeine improve learning and memory in these age groups. Specifically, in young animals, all three treatments—low-dose paraxanthine, high-dose paraxanthine, and caffeine—enhanced learning and memory compared to control. Furthermore, high-dose paraxanthine was found to be more effective than caffeine in improving these cognitive functions in young animals. In old animals, both high-dose paraxanthine and caffeine significantly reduced escape latency compared to control, while a trend was observed for low-dose paraxanthine. Finally, high-dose paraxanthine elevated BDNF levels to a greater extent than caffeine, regardless of age.

Caffeine is a stimulant that, when consumed acutely in high doses, can enhance brain function, and may re-optimize it with chronic moderate consumption, as shown in multi-omic measurements in both rodents (Paiva et al. 2022) and humans (Picó-Pérez et al. 2023). Its primary metabolite, paraxanthine, has been shown to possess similar (Benowitz et al. 1995) or even superior (Orrú et al. 2013) psychoactive properties compared to caffeine. We recently demonstrated that in young adults, 200 mg of paraxanthine, compared to a placebo, can acutely affect short-term memory, reasoning, and response times to cognitive challenges (Yoo et al. 2021), and may serve as an effective nootropic nutrient at an acute dose as low as 50 mg (Xing et al. 2021). In the present study, we aimed to determine if these acute nootropic effects of paraxanthine in humans translate into chronic learning and memory improvements—by directly comparing it with caffeine—while also examining the impact of age and associated biomarker levels. In humans, voluntary caffeine ingestion can occur multiple times daily, whereas in this study, animals were administered caffeine and paraxanthine once a day to assess the effects of a single dose.

Our findings indicate that both high-dose paraxanthine and caffeine supplementation improved learning and memory in both young and old animals, as evidenced by reductions in escape latency. Memory improvements were more pronounced in young animals supplemented with high-dose paraxanthine compared to caffeine. BDNF, a neurotrophin involved in neuronal plasticity (Leal et al. 2017), is essential for learning and memory processes (Radecki et al. 2005). The increased levels of BDNF in animals supplemented with high-dose paraxanthine and caffeine suggest that these compounds may mediate their positive effects on memory. Our results align with previous studies showing that caffeine increases BDNF expression in the hippocampus (Sallaberry et al. 2013), a brain region crucial for learning and memory (Montkowski and Holsboer 1997; Petzold et al. 2015), as well as in a mouse model of Alzheimer’s Disease (Han et al. 2013). Although our measurements were not region-specific, high-dose paraxanthine augmented BDNF levels more than caffeine, suggesting a dose-dependent effect of paraxanthine, regardless of age. Thus, our data indicate that paraxanthine increases BDNF levels to a greater extent than caffeine, which may underlie its cognitive-enhancing effects in both young and old animals. The observed changes in BDNF may explain why paraxanthine showed reduced errors in cognitive flexibility tests (Berg-Wisconsin Card Sorting Test) compared to caffeine (Yoo et al. 2024).

**Fig. 4:**
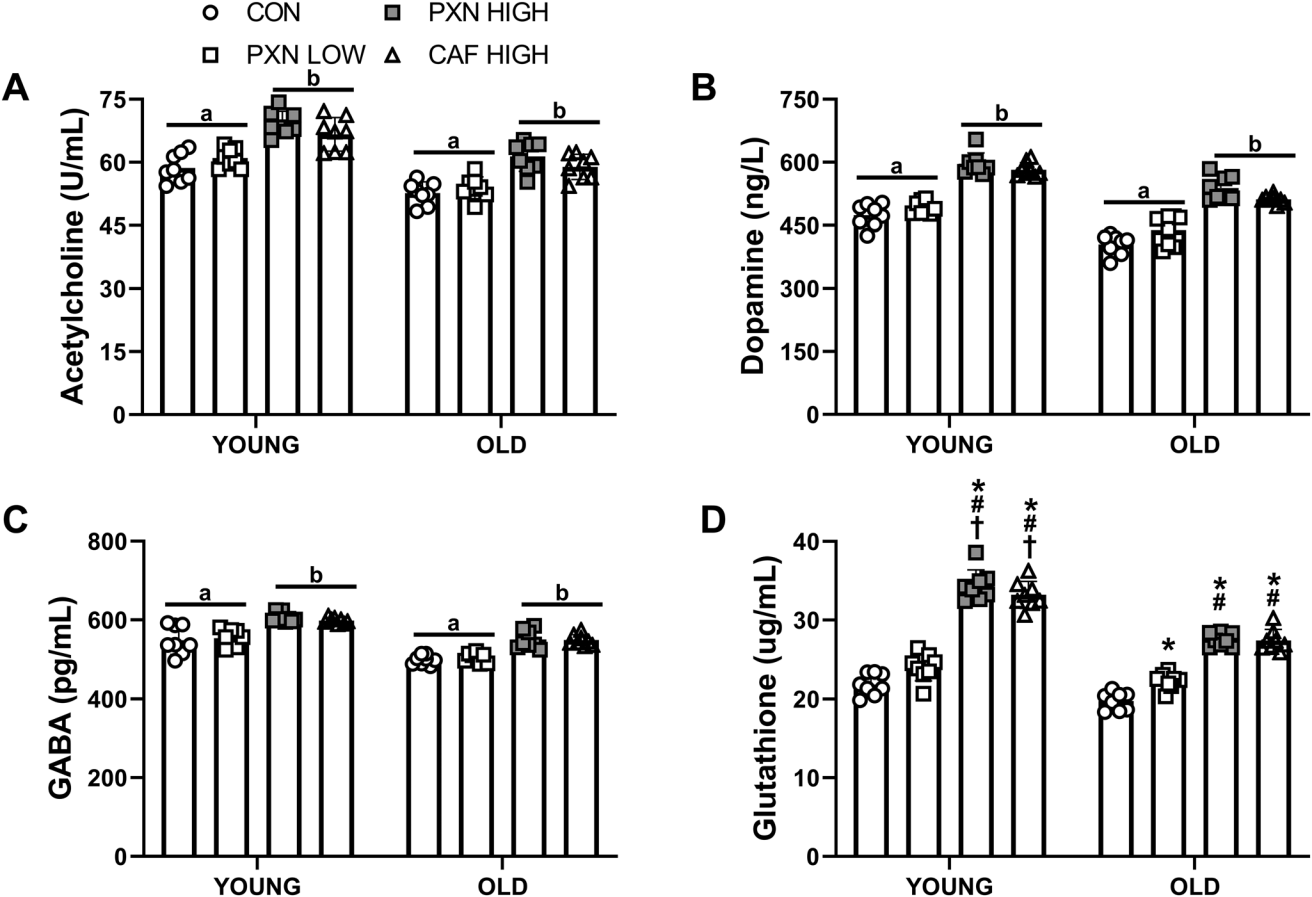
The impact of age and supplementation on (**A**) acetylcholine, dopamine (**B**), GABA (**C**), glutathione (**D**). Letters that are different indicate significantly different group values based on pairwise comparisons following significant group effects (*P* < 0.001).*Different from CON within same age group (*P* < 0.02). #different from PXN LOW within same group (*P* < 0.001). †YOUNG different from OLD within same treatment group (*P* < 0.01). P-values: Group all  < 0.001;; Age all  < 0.001; (**A-C**) Interaction<0.87, (**D**) Interaction<0.001. Data presented as mean ± SD for *n* = 8 in each group. CON, control; PXN LOW, low dose paraxanthine; PXN HIGH, high dose paraxanthine; CAF HIGH, high dose caffeine

It is well established that normal aging is associated with declines in learning and memory (Bettio et al. 2017). Our findings indicate that both paraxanthine and caffeine significantly influence memory, independent of age. In young animals, the greatest improvements were observed with high-dose paraxanthine (PXN HIGH), outperforming all other groups (Fig. 4). In older animals, both high-dose paraxanthine and caffeine (CAF HIGH) produced the most significant improvements, consistent with previous research showing age-related declines in learning and memory acquisition in water maze tests (Shukitt-Hale et al. 2004; de Fiebre et al. 2006). As expected, age significantly affected the levels of all neuromodulators, with young animals exhibiting higher levels than older ones. This aligns with previous studies indicating that aging is associated with decreased levels of BDNF (Lommatzsch et al. 2005; Ziegenhorn et al. 2007), catalase (Tsay et al. 2000), and cyclic GMP (Kelly 2018; Domek-Łopacińska and Strosznajder 2010). Importantly, both PXN HIGH and CAF HIGH were found to increase these neuromodulator levels in both age groups.

Additionally, the reduction in β-amyloid (1–40) levels in animals supplemented with PXN HIGH and CAF HIGH suggests a potential mechanism for the cognitive benefits observed. While our study assessed β-amyloid in the brain, other research has linked β-amyloid accumulation to cognitive impairment and dementia (Näslund et al. 1994; Rodrigue et al. 2009) and shown that high doses of caffeine (e.g., 500 mg) can attenuate these levels (Arendash et al. 2006). Conversely, some studies found that four months of caffeine intake did not significantly influence β-amyloid levels in a transgenic mouse model of Alzheimer’s disease (Stazi et al. 2021). The age-related decline in neuromodulator levels, combined with the lack of significant learning and memory effects from PXN LOW in aged animals (though a trend was noted, *P* = 0.07), may explain the reduced cognitive benefits of both caffeine and paraxanthine in older individuals. Overall, these findings suggest that caffeine and paraxanthine hold therapeutic potential for improving cognitive function, particularly in younger individuals, by enhancing neuromodulation and reducing β-amyloid accumulation.

The present study revealed that both high-dose paraxanthine (PXN HIGH) and high-dose caffeine (CAF HIGH) significantly increased neurotransmitters involved in learning and memory, including acetylcholine, dopamine, and GABA. Acetylcholine is known to be critical for cognitive processes (Hasselmo 2006), and caffeine has been shown to enhance acetylcholine release (Carter et al. 1995) and increase cholinergic neurotransmission (Fabiani et al. 2018). Additionally, dopamine plays a key role in the formation of long-term memories (Rossato et al. 2009; Yamagata et al. 2015), while GABA is involved in the consolidation of these memories (Makkar et al. 2010; Cullen et al. 2014). The modulation of these neurotransmitters by caffeine and paraxanthine may contribute to the observed cognitive improvements. Furthermore, glutathione—an important antioxidant that maintains cellular redox balance (Kalinina et al. 2014) and protects against oxidative damage (Rose et al. 2012)—was found to be elevated in animals supplemented with high doses of paraxanthine and caffeine, irrespective of age. These findings align with previous research indicating that caffeine and its derivatives, including paraxanthine (Matsumura et al. 2023), exert neuroprotective effects by upregulating antioxidant enzymes (Devasagayam et al. 1996) and mitigating oxidative stress-induced cell death (Barcelos et al. 2014).

Notably, the age-related differences in glutathione levels highlight the need for further research to better understand the relationship between age and the effects of paraxanthine and caffeine on glutathione levels and cognition.

Demethylation of caffeine results in the formation of theophylline (1,3-dimethylxanthine), theobromine (3,7-dimethylxanthine), and paraxanthine. The half-life of paraxanthine is approximately 3.1 h, which is shorter than that of caffeine (4.1 h) but significantly different from theophylline (6.2 h) and theobromine (7.2 h) (Nehlig et al., 2018; Lelo et al. 1986). Paraxanthine exhibits lower toxicity than caffeine and is less clastogenic compared to caffeine or theophylline. It is also less harmful in terms of hepatocyte toxicity, less potent as a teratogen than caffeine and theophylline, and less anxiogenic than caffeine (Purpura et al. 2021). Theophylline and theobromine have distinct physiological effects. Theophylline is used as a bronchodilator in asthma treatment (Barnes 2010) and is a more potent adenosine receptor antagonist than caffeine in vitro (Bruns et al. 1983). However, in healthy humans, theophylline does not enhance cognitive function (Fitzpatrick et al. 1992). Theobromine, found in high concentrations in cocoa (Cova et al. 2019), promotes vasodilation and smooth muscle relaxation (Mitchell et al. 2011). Yet, compared to caffeine, clinical studies suggest that theobromine does not significantly influence cognitive processes, such as mood and alertness (Judelson et al. 2013; Mitchell et al. 2011). In summary, while structurally similar dimethylxanthines have different physiological effects, the impact of theophylline and theobromine on cognition is generally less pronounced than that of caffeine and paraxanthine. Direct comparisons between caffeine and its metabolites regarding cognitive effects remain an important area for future research.

The limitations of this study include the absence of measurements for swim speed and distance, as variations in locomotion could influence differences in escape latency. Additionally, we did not assess anxiety levels across the experimental groups. Anxiety is known to impact learning and memory behaviors, and its effects may differ between caffeine and paraxanthine. Moreover, previous research in both mice and rats suggests that the effects of caffeine are more pronounced in preventing memory deterioration rather than enhancing memory in rodents not exposed to potentially harmful stimuli (Cunha et al., 2016). Our findings indicate that control adult rats demonstrated improved learning and memory after caffeine and paraxanthine administration, which may suggest a heightened stress level among these animals. Therefore, future studies should incorporate assessments of anxiety and locomotion to provide a more comprehensive understanding of the effects of these compounds.

## Conclusions

In conclusion, our study provides novel evidence that paraxanthine and caffeine supplementation can enhance learning and memory in both young and old animals, with particularly pronounced effects observed in young animals receiving high-dose paraxanthine. Additionally, paraxanthine may boost cognitive function across age groups by increasing BDNF levels. The observed reduction in β-amyloid levels in animals supplemented with high doses of paraxanthine and caffeine offers further mechanistic insights into their cognitive benefits. The age-related decline in neuromodulators, neurotransmitters, and glutathione levels may account for the diminished cognitive benefits of caffeine and paraxanthine in older animals. These findings underscore the therapeutic potential of paraxanthine for improving cognitive function, especially in younger individuals. Future research is needed to better understand the relationship between age and the effects of paraxanthine and caffeine on cognitive function.

## Electronic supplementary material

Below is the link to the electronic supplementary material.

Supplementary Material 1

## Data Availability

The data generated and analyzed for this current study are available from the corresponding author on reasonable request.

## References

[CR1] Arendash GW, Schleif W, Rezai-Zadeh K, Jackson EK, Zacharia LC, Cracchiolo JR et al (2006) Caffeine protects Alzheimer’s mice against cognitive impairment and reduces brain beta-amyloid production. Neuroscience 142(4):941–952. 10.1016/j.neuroscience.2006.07.021 16938404 10.1016/j.neuroscience.2006.07.021

[CR2] Barcelos RP, Souza MA, Amaral GP, Stefanello ST, Bresciani G, Fighera MR et al (2014) Caffeine supplementation modulates oxidative stress markers in the liver of trained rats. Life Sci 96(1–2):40–45. 10.1016/j.lfs.2013.12.002 24334002 10.1016/j.lfs.2013.12.002

[CR3] Barnes PJ (2010) Theophylline. Pharmaceuticals (Basel). 3(3):725–747. 10.3390/ph3030725 10.3390/ph3030725 PMC4033977 27713276

[CR4] Benowitz NL, Jacob P3, Mayan H, Denaro C (1995) Sympathomimetic effects of paraxanthine and caffeine in humans. Clin Pharmacol Ther 58(6):684–691. 10.1016/0009-9236(95)90025-X 8529334 10.1016/0009-9236(95)90025-X

[CR5] Bettio LEB, Rajendran L, Gil-Mohapel J (2017) The effects of aging in the hippocampus and cognitive decline. Neurosci Biobehav Rev 79:66–86. 10.1016/j.neubiorev.2017.04.030 28476525 10.1016/j.neubiorev.2017.04.030

[CR6] Bhattacharya SK, Satyan KS, Chakrabarti A (1997) Anxiogenic action of caffeine: an experimental study in rats. J Psychopharmacol 11:219–224. 10.1177/026988119701100304 9305413 10.1177/026988119701100304

[CR7] Brockwell NT, Eikelboom R, Beninger RJ (1991) Caffeine-induced place and taste conditioning: production of dose-dependent preference and aversion. Pharmacol Biochem Behav 38:513–517. 10.1016/0091-3057(91)90006-N 2068188 10.1016/0091-3057(91)90006-n

[CR8] Bruns RF, Daly JW, Snyder SH (1983) Adenosine receptor binding: structure-activity analysis generates extremely potent xanthine antagonists. Proc Natl Acad Sci U S A 80(7):2077–2080. 10.1073/pnas.80.7.2077 6300892 10.1073/pnas.80.7.2077 PMC393756

[CR9] Canas PM, Porciúncula LO, Cunha GM, Silva CG, Machado NJ, Oliveira JM et al (2009) Adenosine A2A receptor blockade prevents synaptotoxicity and memory dysfunction caused by beta-amyloid peptides via p38 mitogen-activated protein kinase pathway. J Neurosci 29(47):14741–14751. 10.1523/JNEUROSCI.3728-09.2009 19940169 10.1523/JNEUROSCI.3728-09.2009 PMC6665997

[CR10] Carter AJ, O’Connor WT, Carter MJ, Ungerstedt U (1995) Caffeine enhances acetylcholine release in the hippocampus in vivo by a selective interaction with adenosine A1 receptors. J Pharmacol Exp Ther 273(2):637–642 7752065

[CR11] Cognato GP, Agostinho PM, Hockemeyer J, Müller CE, Souza DO, Cunha RA (2010) Caffeine and an adenosine A(2A) receptor antagonist prevent memory impairment and synaptotoxicity in adult rats triggered by a convulsive episode in early life. J Neurochem 112(2):453–462. 10.1111/j.1471-4159.2009.06465.x 19878534 10.1111/j.1471-4159.2009.06465.x

[CR12] Cova I, Leta V, Mariani C, Pantoni L, Pomati S (2019) Exploring cocoa properties: is theobromine a cognitive modulator? Psychopharmacology 236(2):561–572. 10.1007/s00213-019-5172-0 30706099 10.1007/s00213-019-5172-0

[CR14] Culig L, Chu X, Bohr VA (2022) Neurogenesis in aging and age-related neurodegenerative diseases. Ageing Res Rev 78:101636. 10.1016/j.arr.2022.101636 35490966 10.1016/j.arr.2022.101636 PMC9168971

[CR13] Cullen PK, Dulka BN, Ortiz S, Riccio DC, Jasnow AM (2014) GABA-mediated presynaptic inhibition is required for precision of long-term memory. Learn Mem 21(4):180–184. 10.1101/lm.032961.113 24634352 10.1101/lm.032961.113 PMC3966537

[CR15] Cunha RA (2016) How does adenosine control neuronal dysfunction and neurodegeneration? J Neurochem 139(6):1019–1055. 10.1111/jnc.13724 27365148 10.1111/jnc.13724

[CR16] Dall’Igna OP, Porciúncula LO, Souza DO, Cunha RA, Lara DR (2003) Neuroprotection by caffeine and adenosine A2A receptor blockade of beta-amyloid neurotoxicity. Br J Pharmacol 138(7):1207–1209. 10.1038/sj.bjp.0705185 12711619 10.1038/sj.bjp.0705185 PMC1573785

[CR17] de Fiebre NC, Sumien N, Forster MJ, de Fiebre CM (2006) Spatial learning and psychomotor performance of C57BL/6 mice: age sensitivity and reliability of individual differences. Age (Dordr) 28(3):235–253. 10.1007/s11357-006-9027-3 22253492 10.1007/s11357-006-9027-3 PMC3259155

[CR19] deToledo-Morrell L, Geinisman Y, Morrell F (1988) Age-dependent alterations in hippocampal synaptic plasticity: relation to memory disorders. Neurobiol Aging 9(5–6):581–590. 10.1016/s0197-4580(88)80117-9 3062469 10.1016/s0197-4580(88)80117-9

[CR18] Devasagayam TP, Kamat JP, Mohan H, Kesavan PC (1996) Caffeine as an antioxidant: inhibition of lipid peroxidation induced by reactive oxygen species. Biochim Biophys Acta 1282(1):63–70. 10.1016/0005-2736(96)00040-5 8679661 10.1016/0005-2736(96)00040-5

[CR20] Domek-Łopacińska KU, Strosznajder JB (2010) Cyclic GMP and nitric oxide synthase in aging and Alzheimer’s disease. Mol Neurobiol 41(2–3):129–137. 10.1007/s12035-010-8104-x 20213343 10.1007/s12035-010-8104-x

[CR21] Duarte JM, Agostinho PM, Carvalho RA, Cunha RA (2012) Caffeine consumption prevents diabetes-induced memory impairment and synaptotoxicity in the hippocampus of NONcZNO10/LTJ mice. PLoS ONE 7(4):e21899. 10.1371/journal.pone.0021899 22514596 10.1371/journal.pone.0021899 PMC3326010

[CR22] Eskelinen MH, Kivipelto M (2010) Caffeine as a protective factor in dementia and Alzheimer’s disease. J Alzheimers Dis 20(Suppl 1):S167–S174. 10.3233/JAD-2010-1404 20182054 10.3233/JAD-2010-1404

[CR23] Espinosa J, Rocha A, Nunes F, Costa MS, Schein V, Kazlauckas V et al (2013) Caffeine consumption prevents memory impairment, neuronal damage, and adenosine A2A receptors upregulation in the hippocampus of a rat model of sporadic dementia. J Alzheimers Dis 34(2):509–518. 10.3233/JAD-111982 23241554 10.3233/JAD-111982

[CR24] Fabiani C, Murray AP, Corradi J, Antollini SS (2018) A novel pharmacological activity of caffeine in the cholinergic system. Neuropharmacology 135:464–473. 10.1016/j.neuropharm.2018.03.041 29614315 10.1016/j.neuropharm.2018.03.041

[CR25] Ferré S, Orrú M, Guitart X (2013) Paraxanthine: connecting caffeine to nitric oxide neurotransmission. J Caffeine Res 3(2):72–78. 10.1089/jcr.2013.0006 24761277 10.1089/jcr.2013.0006 PMC3680978

[CR26] Fitzpatrick MF, Engleman HM, Boellert F, McHardy R, Shapiro CM, Deary IJ, Douglas NJ (1992) Effect of therapeutic theophylline levels on the sleep quality and daytime cognitive performance of normal subjects. Am Rev Respir Dis 145(6):1355–1358. 10.1164/ajrccm/145.6.1355 1596003 10.1164/ajrccm/145.6.1355

[CR27] Gonçalves FQ, Lopes JP, Silva HB, Lemos C, Silva AC, Gonçalves N et al (2019) Synaptic and memory dysfunction in a β-amyloid model of early Alzheimer’s disease depends on increased formation of ATP-derived extracellular adenosine. Neurobiol Dis 132:104570. 10.1016/j.nbd.2019.104570 31394204 10.1016/j.nbd.2019.104570

[CR28] Guerreiro S, Toulorge D, Hirsch E, Marien M, Sokoloff P, Michel PP (2008) Paraxanthine, the primary metabolite of caffeine, provides protection against dopaminergic cell death via stimulation of ryanodine receptor channels. Mol Pharmacol 74(4):980–989. 10.1124/mol.108.048207 18621927 10.1124/mol.108.048207

[CR29] Guest N, Corey P, Vescovi J, El-Sohemy A (2018) Caffeine, CYP1A2 genotype, and endurance performance in athletes. Med Sci Sports Exerc 50(8):1570–1578. 10.1249/MSS.0000000000001596 29509641 10.1249/MSS.0000000000001596

[CR30] Guest NS, VanDusseldorp TA, Nelson MT, Grgic J, Schoenfeld BJ, Jenkins NDM et al (2021) International society of sports nutrition position stand: caffeine and exercise performance. J Int Soc Sports Nutr 18(1):1. 10.1186/s12970-020-00383-4 33388079 10.1186/s12970-020-00383-4 PMC7777221

[CR31] Han K, Jia N, Li J, Yang L, Min L (2013) Chronic caffeine treatment reverses memory impairment and the expression of brain BNDF and TrkB in the PS1/APP double transgenic mouse model of Alzheimer’s disease. Mol Med Rep 8:737–740. 10.3892/mmr.2013.1601 23900282 10.3892/mmr.2013.1601 PMC3782531

[CR32] Hasselmo ME (2006) The role of acetylcholine in learning and memory. Curr Opin Neurobiol 16(6):710–715. 10.1016/j.conb.2006.09.002 17011181 10.1016/j.conb.2006.09.002 PMC2659740

[CR33] Judelson DA, Preston AG, Miller DL, Muñoz CX, Kellogg MD, Lieberman HR (2013) Effects of theobromine and caffeine on mood and vigilance. J Clin Psychopharmacol 33(4):499–506. 10.1097/JCP.0b013e3182905d24 23764688 10.1097/JCP.0b013e3182905d24

[CR35] Kalinina EV, Chernov NN, Novichkova MD (2014) Role of glutathione, glutathione transferase, and glutaredoxin in regulation of redox-dependent processes. Biochem (Mosc) 79(13):1562–1583. 10.1134/S0006297914130082 10.1134/S0006297914130082 25749165

[CR34] Kaplan GB, Greenblatt DJ, Ehrenberg BL, Goddard JE, Cotreau MM, Harmatz JS, Shader RI (1997) (dose-dependent pharmacokinetics and psychomotor effects of caffeine in humans. J Clin Pharmacol 37(8):693–703. 10.1002/j.1552-4604.1997.tb04356.x 9378841 10.1002/j.1552-4604.1997.tb04356.x

[CR36] Kaster MP, Machado NJ, Silva HB, Nunes A, Ardais AP, Santana M et al (2015) Caffeine acts through neuronal adenosine A2A receptors to prevent mood and memory dysfunction triggered by chronic stress. Proc Natl Acad Sci U S A. 23;112(25):7833-8. 10.1073/pnas.1423088112 10.1073/pnas.1423088112 PMC4485143 26056314

[CR37] Kelly MP (2018) Cyclic nucleotide signaling changes associated with normal aging and age-related diseases of the brain. Cell Signal 42:281–291. 10.1016/j.cellsig.2017.11.004 29175000 10.1016/j.cellsig.2017.11.004 PMC5732030

[CR38] Laurent C, Eddarkaoui S, Derisbourg M, Leboucher A, Demeyer D, Carrier S et al (2014) Beneficial effects of caffeine in a transgenic model of Alzheimer’s disease-like tau pathology. Neurobiol Aging 35(9):2079–2090. 10.1016/j.neurobiolaging.2014.03.027 24780254 10.1016/j.neurobiolaging.2014.03.027

[CR39] Leal G, Bramham CR, Duarte CB (2017) BDNF and hippocampal synaptic plasticity. Vitam Horm 104:153–195. 10.1016/bs.vh.2016.10.004 28215294 10.1016/bs.vh.2016.10.004

[CR40] Lelo A, Birkett DJ, Robson RA, Miners JO (1986) Comparative pharmacokinetics of caffeine and its primary demethylated metabolites paraxanthine, theobromine and theophylline in man. Br J Clin Pharmacol 22(2):177–182. 10.1111/j.1365-2125.1986.tb05246.x 3756065 10.1111/j.1365-2125.1986.tb05246.x PMC1401099

[CR41] Lieberman HR, Wurtman RJ, Emde GG, Roberts C, Coviella IL (1987) The effects of low doses of caffeine on human performance and mood. Psychopharmacology 92(3):308–312. 10.1007/BF00210835 3114783 10.1007/BF00210835

[CR42] Lommatzsch M, Zingler D, Schuhbaeck K, Schloetcke K, Zingler C, Schuff-Werner P et al (2005) The impact of age, weight and gender on BDNF levels in human platelets and plasma. Neurobiol Aging 26(1):115–123. 10.1016/j.neurobiolaging.2004.03.002 15585351 10.1016/j.neurobiolaging.2004.03.002

[CR43] Makkar SR, Zhang SQ, Cranney J (2010) Behavioral and neural analysis of GABA in the acquisition, consolidation, reconsolidation, and extinction of fear memory. Neuropsychopharmacology 35(8):1625–1652. 10.1038/npp.2010.53 20410874 10.1038/npp.2010.53 PMC3055480

[CR44] Matsumura N, Kinoshita C, Bhadhprasit W, Nakaki T, Aoyama K (2023) A purine derivative, paraxanthine, promotes cysteine uptake for glutathione synthesis. J Pharmacol Sci 151(1):37–45. 10.1016/j.jphs.2022.11.001 36522121 10.1016/j.jphs.2022.11.001

[CR45] Minaei S, Rahimi MR, Mohammadi H, Jourkesh M, Kreider RB et al (2022) CYP1A2 genotype polymorphism influences the Effect of Caffeine on anaerobic performance in trained males. Int J Sport Nutr Exerc Metab 32(1):16–21. 10.1123/ijsnem.2021-0090 34611052 10.1123/ijsnem.2021-0090

[CR46] Mitchell ES, Slettenaar M, vd Meer N, Transler C, Jans L, Quadt F, Berry M (2011) Differential contributions of theobromine and caffeine on mood, psychomotor performance and blood pressure. Physiol Behav 104(5):816–822. 10.1016/j.physbeh.2011.07.027 21839757 10.1016/j.physbeh.2011.07.027

[CR47] Montkowski A, Holsboer F (1997) Intact spatial learning and memory in transgenic mice with reduced BDNF. NeuroReport 8(3):779–782. 10.1097/00001756-199702100-00040 9106766 10.1097/00001756-199702100-00040

[CR48] Mora F, Segovia G, del Arco A (2007) Aging, plasticity and environmental enrichment: structural changes and neurotransmitter dynamics in several areas of the brain. Brain Res Rev 55(1):78–88. 10.1016/j.brainresrev.2007.03.011 17561265 10.1016/j.brainresrev.2007.03.011

[CR49] Morris R (1984) Developments of a water-maze procedure for studying spatial learning in the rat. J Neurosci Methods 11(1):47–60. 10.1016/0165-0270(84)90007-4 6471907 10.1016/0165-0270(84)90007-4

[CR50] Nair AB, Jacob S (2016) A simple practice guide for dose conversion between animals and human. J Basic Clin Pharm 7(2):27–31. 10.4103/0976-0105.177703 27057123 10.4103/0976-0105.177703 PMC4804402

[CR51] Näslund J, Schierhorn A, Hellman U, Lannfelt L, Roses AD, Tjernberg LO et al (1994) Relative abundance of Alzheimer A beta amyloid peptide variants in Alzheimer disease and normal aging. *Proc. Natl. Acad. Sci. U. S. A.* 91(18);8378–8382. 10.1073/pnas.91.18.8378 10.1073/pnas.91.18.8378 PMC44609 8078890

[CR52] Nehlig A (2018) Interindividual Differences in Caffeine Metabolism and factors driving caffeine consumption. Pharmacol Rev 70(2):384–411. 10.1124/pr.117.014407 29514871 10.1124/pr.117.014407

[CR53] Nunez J (2008) Morris Water Maze Experiment. J Vis Exp 19:897. 10.3791/897 10.3791/897 PMC2872979 19066539

[CR54] Orrú M, Guitart X, Karcz-Kubicha M, Solinas M, Justinova Z, Barodia SK et al (2013) Psychostimulant pharmacological profile of paraxanthine, the main metabolite of caffeine in humans. Neuropharmacology 67:476–484. 10.1016/j.neuropharm.2012.11.029 23261866 10.1016/j.neuropharm.2012.11.029 PMC3562388

[CR55] Ozawa T, Kaseda K, Ichitani Y, Yamada K (2022) Caffeine facilitates extinction of auditory fear conditioning in rats. Neuropsychopharmacol Rep 42:521–525. 10.1002/npr2.12287 35960195 10.1002/npr2.12287 PMC9773722

[CR56] Paiva I, Cellai L, Meriaux C, Poncelet L, Nebie O, Saliou JM et al (2022) Caffeine intake exerts dual genome-wide effects on hippocampal metabolism and learning-dependent transcription. J Clin Invest 132(12):e149371. 10.1172/JCI149371 35536645 10.1172/JCI149371 PMC9197525

[CR57] Pataky MW, Womack CJ, Saunders MJ, Goffe JL, D’Lugos AC, El-Sohemy A et al (2016) Caffeine and 3-km cycling performance: effects of mouth rinsing, genotype, and time of day. Scand J Med Sci Sports 26(6):613–619. 10.1111/sms.12501 26062916 10.1111/sms.12501

[CR58] Petzold A, Psotta L, Brigadski T, Endres T, Lessmann V (2015) Chronic BDNF deficiency leads to an age-dependent impairment in spatial learning. Neurobiol Learn Mem 120:52–60. 10.1016/j.nlm.2015.02.009 25724412 10.1016/j.nlm.2015.02.009

[CR59] Picó-Pérez M, Magalhães R, Esteves M, Vieira R, Castanho TC, Amorim L et al (2023) Coffee consumption decreases the connectivity of the posterior default Mode Network (DMN) at rest. Front Behav Neurosci 17:1176382. 10.3389/fnbeh.2023.1176382 37448789 10.3389/fnbeh.2023.1176382 PMC10336217

[CR60] Purpura M, Jäger R, Falk M (2021) An assessment of mutagenicity, genotoxicity, acute-, subacute and subchronic oral toxicity of paraxanthine (1,7-dimethylxanthine). Food Chem Toxicol 158:112579. 10.1016/j.fct.2021.112579 34597720 10.1016/j.fct.2021.112579

[CR61] Radecki DT, Brown LM, Martinez J, Teyler TJ (2005) BDNF protects against stress-induced impairments in spatial learning and memory and LTP. Hippocampus 152:246–253 10.1002/hipo.20048 15476265

[CR62] Rees K, Allen D, Lader M (1999) The influences of age and caffeine on psychomotor and cognitive function. Psychopharmacology 145(2):181–188. 10.1007/s002130051047 10463319 10.1007/s002130051047

[CR63] Rodrigue KM, Kennedy KM, Park DC (2009) Beta-amyloid deposition and the aging brain. Neuropsychol Rev 19(4):436–450. 10.1007/s11065-009-9118-x 19908146 10.1007/s11065-009-9118-x PMC2844114

[CR64] Rose S, Melnyk S, Pavliv O, Bai S, Nick TG, Frye RE et al (2012) Evidence of oxidative damage and inflammation associated with low glutathione redox status in the autism brain. Transl Psychiatry 2(7):e134. 10.1038/tp.2012.61 22781167 10.1038/tp.2012.61 PMC3410618

[CR65] Rossato JI, Bevilaqua LRM, Izquierdo I, Medina JH, Cammarota M (2009) Dopamine controls persistence of long-term memory storage. Science 325(5943):1017–1020. 10.1126/science.1172545 19696353 10.1126/science.1172545

[CR66] Ryan L, Hatfield C, Hofstetter M (2002) Caffeine reduces time-of-day effects on memory performance in older adults. Psychol Sci 13(1):68–71. 10.1111/1467-9280.00412 11892781 10.1111/1467-9280.00412

[CR67] Sallaberry C, Nunes F, Costa MS, Fioreze GT, Ardais AP, Botton PHS et al (2013) Chronic caffeine prevents changes in inhibitory avoidance memory and hippocampal BDNF immunocontent in middle-aged rats. Neuropharmacology 64:153–159. 10.1016/j.neuropharm.2012.07.010 22841916 10.1016/j.neuropharm.2012.07.010

[CR68] Shukitt-Hale B, McEwen JJ, Szprengiel A, Joseph JA (2004) Effect of age on the radial arm water maze-a test of spatial learning and memory. Neurobiol Aging 25(2):223–229. 10.1016/s0197-4580(03)00041-1 14749140 10.1016/s0197-4580(03)00041-1

[CR70] Stavric B (1988) Methylxanthines: toxicity to humans. 3. Theobromine, paraxanthine and the combined effects of methylxanthines. Food Chem Toxicol 26(8):725–733. 10.1016/0278-6915(88)90073-7 3058562 10.1016/0278-6915(88)90073-7

[CR69] Stazi M, Lehmann S, Sakib MS, Pena-Centeno T, Büschgens L, Fischer A et al (2021) Long-term caffeine treatment of Alzheimer mouse models ameliorates behavioural deficits and neuron loss and promotes cellular and molecular markers of neurogenesis. Cell Mol Life Sci 79(1):55. 10.1007/s00018-021-04062-8 34913091 10.1007/s00018-021-04062-8 PMC8738505

[CR71] Temido-Ferreira M, Ferreira DG, Batalha VL, Marques-Morgado I, Coelho JE et al (2020) Age-related shift in LTD is dependent on neuronal adenosine A_2A_ receptors interplay with mGluR5 and NMDA receptors. Mol Psychiatry 25(8):1876–1900. 10.1038/s41380-018-0110-9 29950682 10.1038/s41380-018-0110-9 PMC7387321

[CR72] Tsay HJ, Wang P, Wang SL, Ku HH (2000) Age-associated changes of superoxide dismutase and catalase activities in the rat brain. J Biomed Sci 7(6):466–474. 10.1007/BF02253362 11060495 10.1007/BF02253362

[CR73] Viana da Silva S, Haberl MG, Zhang P, Bethge P, Lemos C, Gonçalves N et al (2016) Early synaptic deficits in the APP/PS1 mouse model of Alzheimer’s disease involve neuronal adenosine A2A receptors. Nat Commun 7:11915. 10.1038/ncomms11915 27312972 10.1038/ncomms11915 PMC4915032

[CR74] Womack CJ, Saunders MJ, Bechtel MK, Bolton DJ, Martin M, Luden ND et al (2012) The influence of a CYP1A2 polymorphism on the ergogenic effects of caffeine. J Int Soc Sports Nutr 9(1):7. 10.1186/1550-2783-9-7 22420682 10.1186/1550-2783-9-7 PMC3334681

[CR75] Xing D, Yoo C, Gonzalez D, Jenkins V, Nottingham K, Dickerson B et al (2021) Dose-response of Paraxanthine on cognitive function: a double blind, placebo controlled, crossover trial. Nutrients 13(12):4478. 10.3390/nu13124478 34960030 10.3390/nu13124478 PMC8708375

[CR77] Yamagata N, Ichinose T, Aso Y, Plaçais P, Friedrich AB, Sima RJ et al (2015) Distinct dopamine neurons mediate reward signals for short- and long-term memories. Proc Natl Acad Sci U S A 112(2):578–583. 10.1073/pnas.1421930112 25548178 10.1073/pnas.1421930112 PMC4299218

[CR78] Yoo C, Xing D, Gonzalez D, Jenkins V, Nottingham K, Dickerson B et al (2021) Acute Paraxanthine Ingestion improves cognition and short-term memory and helps sustain attention in a Double-Blind, Placebo-Controlled, crossover trial. Nutrients 13(11):3980. 10.3390/nu13113980 34836235 10.3390/nu13113980 PMC8622427

[CR76] Yoo C, Xing D, Gonzalez DE, Jenkins V, Nottingham K, Dickerson B et al (2024) Paraxanthine provides greater improvement in cognitive function than caffeine after performing a 10-km run. J Int Soc Sports Nutr 21(1):2352779. 10.1080/15502783.2024.2352779 38725238 10.1080/15502783.2024.2352779 PMC11089923

[CR79] Ziegenhorn AA, Schulte-Herbrüggen O, Danker-Hopfe H, Malbranc M, Hartung H, Anders D et al (2007) Serum neurotrophins–a study on the time course and influencing factors in a large old age sample. Neurobiol Aging 28(9):1436–1445. 10.1016/j.neurobiolaging.2006.06.011 16879899 10.1016/j.neurobiolaging.2006.06.011

