# Correction: Paraxanthine enhances memory and neuroplasticity more than caffeine in rats

**DOI:** 10.1007/s00221-025-06996-y

**Published:** 2025-02-05

**Authors:** Ralf Jäger, Sidney Abou Sawan, Marco Orrú, Grant M. Tinsley, Martin Purpura, Shawn D. Wells, Kylin Liao, Ashok Godavarthi

**Affiliations:** 1 Ingenious Ingredients L.P, Lewisville, TX 75056 USA; 2 Increnovo LLC, Whitefish Bay, WI 53217 USA; 3 Iovate Health Sciences International, Oakville, ON L6M 0H4 Canada; 4 Department of Pharmacology and Toxicology, University of Utah, Salt Lake City, UT USA; 5 Department of Kinesiology and Sport Management, Texas Tech University, Lubbock, TX USA; 6 Radiant Research Services Pvt. Ltd, Bangalore, 560058 India

**Correction: Experimental Brain Research (2025) 243:8** 10.1007/s00221-024-06954-0

In the original version of this article, the given and family names of Sidney Abou Sawan were incorrectly structured. The name was displayed correctly in all versions at the time of publication.

In addition, in this article, the caption to Fig. 2 was inadvertently swapped to Fig. 3, the caption to Fig. 3 was inadvertently swapped to Fig. 4 and the caption to Fig. 4 was inadvertently swapped to Fig. 2.

In this article the wrong figure appeared as Fig. 1; the figure should have appeared as shown below.

Incorrect Version of Fig. 1:
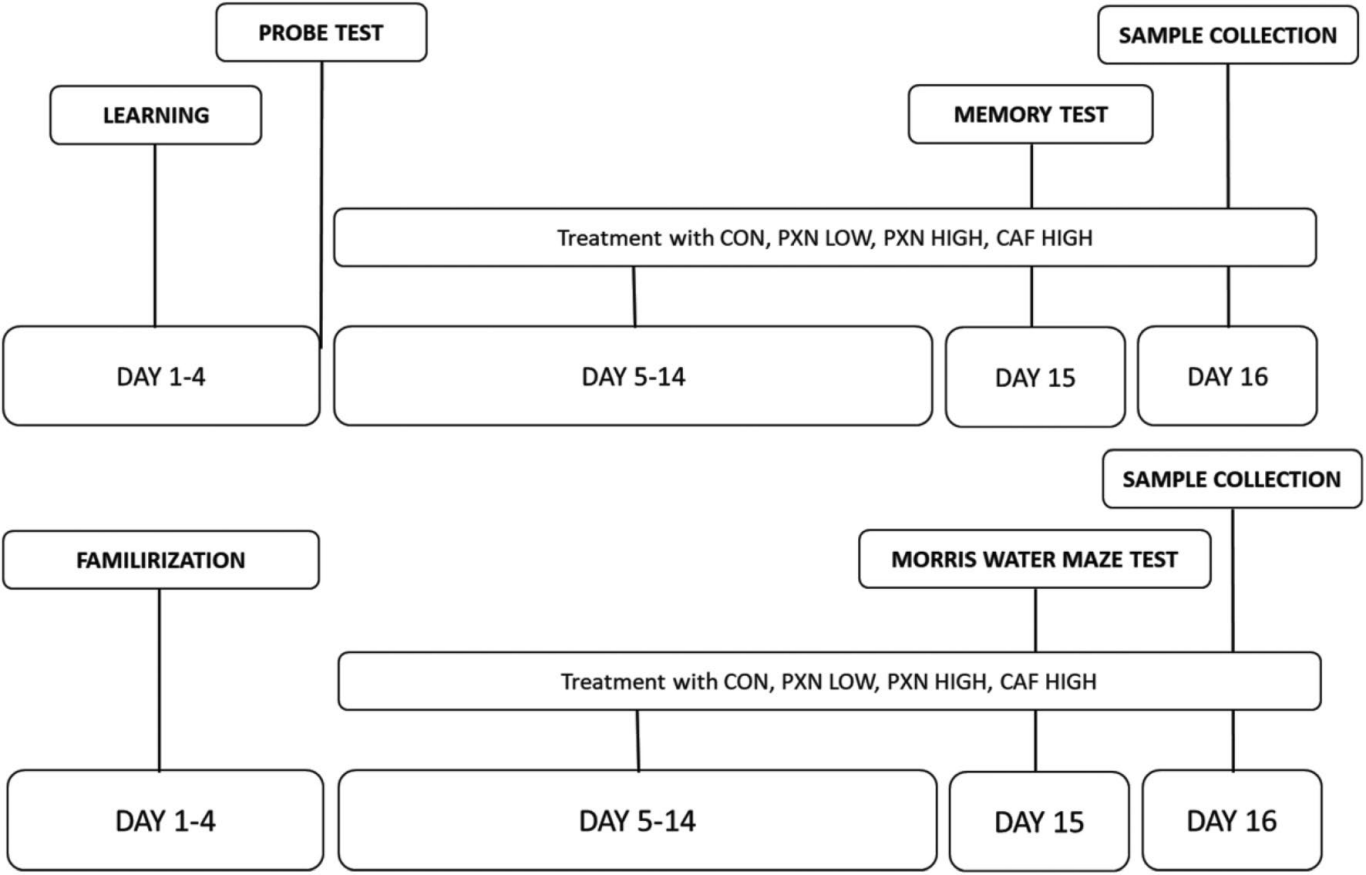


Correct Version of Fig. 1:
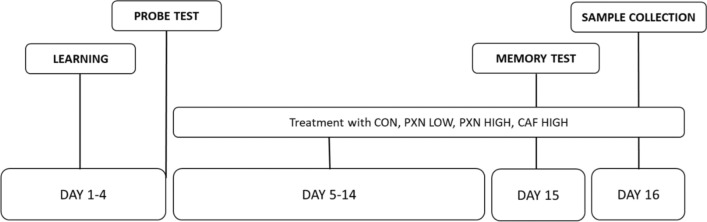


The original article has been corrected.

